# Placenta-derived mesenchymal stem cells possess better immunoregulatory properties compared to their cord-derived counterparts–a paired sample study

**DOI:** 10.1038/srep15784

**Published:** 2015-10-28

**Authors:** Manasi D. Talwadekar, Vaijayanti P. Kale, Lalita S. Limaye

**Affiliations:** 1 Stem Cell Laboratory, National Centre for Cell Science, NCCS Complex, University of Pune Campus, Ganeshkhind, Pune 411007, India

## Abstract

Mesenchymal stem cells (MSCs) show immunoregulatory properties. Here, we compared MSCs obtained from placenta (P-MSCs) and umbilical cord (C-MSCs) from the same donor, for their immunomodulatory efficacy. P-MSCs and C-MSCs showed similar morphology and phenotypic profile, but different clonogenic ability. Importantly, they showed a significant difference in their immunosuppressive properties as assessed in mixed leukocyte reaction (MLR). The P-MSCs affected the antigen presenting ability of mononuclear cells (MNCs) and dendritic cells (DCs) significantly as compared to C-MSCs resulting in a reduced T-cell proliferation. P-MSC conditioned medium (CM) showed a significant reduction in T cell proliferation as compared to C-MSC CM, thus suggesting that a cell to cell contact is not essential. We found increased levels of IL-10 and TGFβ1 and reduction in levels of IFNγ in P-MSC MLRs as compared to C-MSC MLRs. Furthermore, the CD3^+^ CD4^+^ CD25^+^ T regulatory cells were enriched in case of P-MSCs in both, MSC-MNC and MSC-DC co-cultures. This observation was further supported by increased mRNA expression of FoxP3 in P-MSCs. Presently, cord-derived MSCs are being employed in transplantation therapies parallel to the bone marrow-derived MSCs. Our findings suggest that P-MSCs can be a better alternative to C-MSCs, to provide aid in immunological ailments.

Mesenchymal stem cells (MSCs) belong to the category of adult stem cells, of the non-haematopoietic lineage, found to be resident in many tissues, where they act as a pool of self renewing cells which can differentiate into desired cell type after a tissue injury^1^,^2^. These cells, isolated and identified first by Friedenstein from bone marrow (BM)^3^, are best known for their proliferative and mesodermal lineage differentiation ability on the basis of which they are now been used in many tissue repair regimes. Although isolation of MSCs has been successfully achieved from other tissues, such as adipose tissue, gingiva, placenta, umbilical cord, etc.^2^,^4^,^5^, variations in terms of their extent of proliferation and behaviour have been reported. Among all the sources, the umbilical cord tissues have the least ethical constraints being majorly clinical wastes and involve no invasive method for procurement. The beneficial effect of MSCs in alleviating the diseased state is attributed to their cytokine secretion, migration ability and the immunomodulatory function. Their immune regulatory properties have been evaluated in animal models of multiple sclerosis^6^,^7^ and rheumatoid arthritis^8^,^9^, where the impact is on the cells of immune system. MSCs exert regulatory effects on various cells of immune system such as dendritic cells, NK cells and T cells^10^,^11^,^12^. It is well established that MSCs lack the MHC class II molecule and hence do not mount an immune response; but instead they secrete cytokines such as prostaglandin E2 (PGE2), Interleukin 10 (IL-10), Interleukin 6 (IL-6), transforming growth factor β (TGFβ), hepatocyte growth factor (HGF) etc. that are known to be involved in anti-inflammatory responses^13^,^14^,^15^. The effect of MSCs is seen on the maturation of antigen presenting cells, wherein they downregulate the expression of co-stimulatory molecules, thereby affecting the immunogenic response^12^,^16^. These properties have been successfully used in graft versus host disease (GVHD)^17^,^18^ in many clinical trials along with other immune-related diseases. However, in all the studies reported, bone marrow-derived MSCs from allogenic sources are used and umbilical cord tissue-derived MSCs are now being introduced^19^. Studies with bone marrow and umbilical cord derived MSCs have looked into their immunosuppressive properties with a concomitant increase in the regulatory T cell fraction after MSC administration^20^,^21^. But a source-dependent variation in the behaviour of MSCs has been observed. Here, we compared the immunomodulatory potential of MSCs derived from placenta and umbilical cord, obtained from the same individual.

Very few reports talk about the source dependent analysis of the effect of MSCs on the immune cells, where the comparison of sources is from different donors^22^. The donor variation in this context cannot be neglected suggesting a need to isolate MSCs from different sources obtained from the same donor^23^. To study this effect, we co-cultured MSCs derived from human umbilical cord and placenta either with mononuclear cells or with dendritic cells. The effect mounted by this co-culture on the T cells in a mixed leukocyte reaction (MLR) was then assessed. We looked into the enrichment of any specific T cell subset in the MLR due to the presence of MSCs. We went on to examine the regulatory milieu by analyzing the cytokine profile of the MLRs.

We report here that P-MSCs bring about higher reduction in T cell proliferation in both types of MLRs compared to C-MSCs, and this is mainly due to the enrichment of regulatory T cell subset. A cell to cell contact is not necessary as even the CMs from the two types of MSCs showed a similar effect. Thus, by using paired samples – to minimize the sample variation and define the condition set of the isolated tissues – we show for the first time conclusively that there are striking differences in the two types of MSCs in their immunomodulatory behaviour. This study will help investigators to identify the proper source for MSCs in treatment of conditions like GVHD.

## Results

### Placenta- and umbilical cord-derived MSCs from paired samples showed similar phenotype, but different clonogenic ability

**Figure 1:**
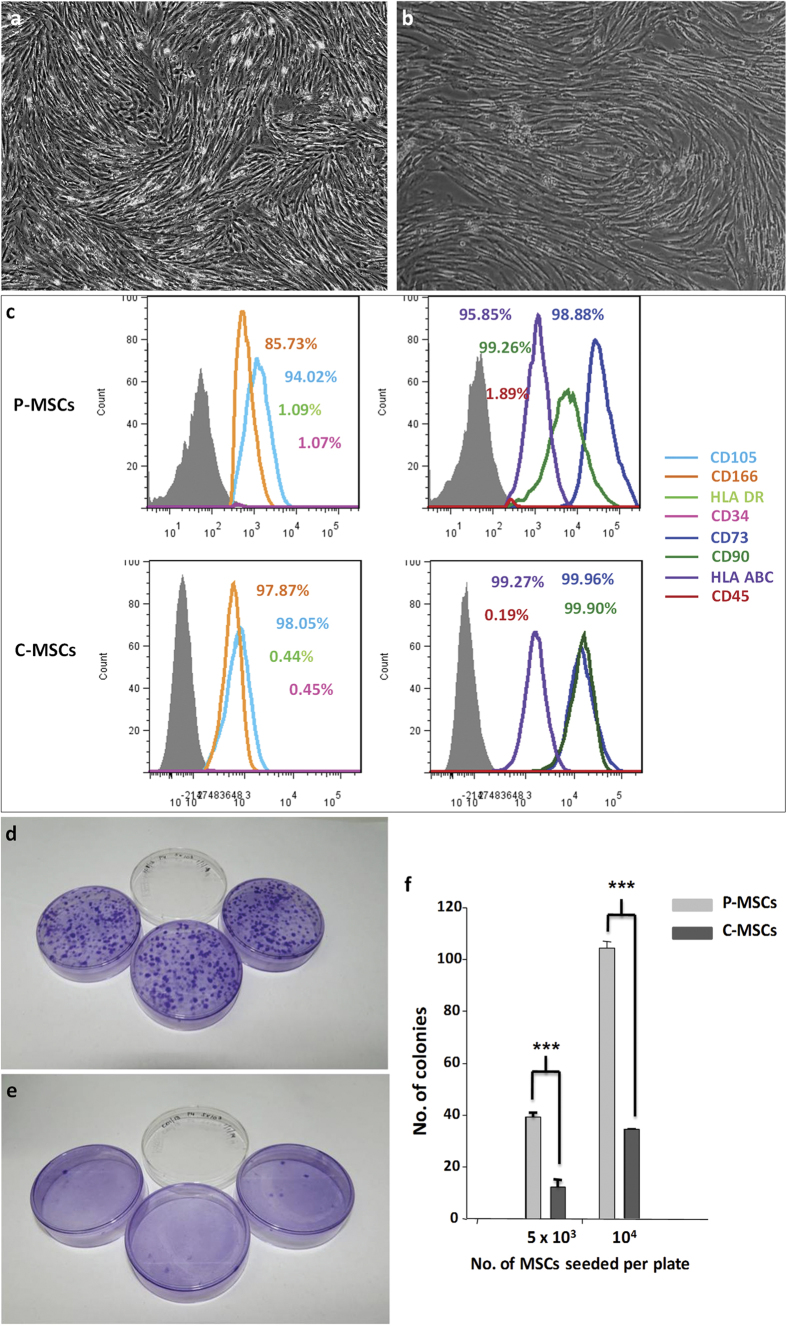
P-MSCs and C-MSCs exhibit similar morphology and phenotype. MSCs isolated from (**a**) placenta and (**b**) umbilical cord showed typical fibroblastic morphology. Phenotypic characterization of (**c**) placental and cord MSCs showing positive expression of CD73, CD105, CD166, CD90, HLA ABC. The cells were negative for haematopoietic stem cell marker CD34, pan leukocyte marker CD45 and MHC class II molecule HLA DR in their respective overlays with the percentage of expression. The key to the overlays is as indicated with the image. Colony forming unit fibroblast – CFU F assay for clonogenecity - extent of colony formation where colonies stained with crystal violet were scored as in (**d**) P-MSCs and (**e**) C-MSCs with (**f**) graph showing the statistical difference between the two MSCs after seeding 5 × 10^3^ cells and 10^4^ cells per plate.

The MSCs derived from placenta and umbilical cord displayed typical fibroblastic morphology [Fig. 1a,b]. They also showed a similar marker expression profile, where they showed more than 90% expression for CD73, CD105, CD166, CD90, MHC class I molecule HLA ABC and were negative for expression of CD34, CD45, and MHC class II molecule HLA DR [Fig. 1c,d]. Due to the absence of MHC class II molecule, MSCs are reported to exhibit immunomodulatory abilities which grants these cells an advantage in transplantation settings^12^. Both the MSCs showed mesodermal lineage differentiation ability when subjected to adipocytic, osteocytic and chondrogenic differentiation as assessed by the Oil Red O staining, Alizarin Red S and Alcian blue staining respectively [Supplementary Fig. S1]. The clonogenic nature of a cell is defined by its ability to give rise to a colony of cells; this property is shown to be exhibited by MSCs which was assessed by the colony forming unit – fibroblast (CFU-F) assay. Plating of MSCs at different cell concentrations and then scoring for the number of colonies generated after 7–10 days showed that the clonogenic ability of P-MSCs and C-MSCs was different, though the tissues were isolated from the same individual. The colonies formed were assessed after 10 days by staining with crystal violet staining solution where the number of colonies in case of P-MSCs [Fig. 1e,g] were significantly more as compared to those in case of C-MSCs [Fig. 1f,g]. This suggests a higher expansion ability of P-MSCs.

### P-MSCs have higher immunosuppressive ability compared to C-MSCs

**Figure 2:**
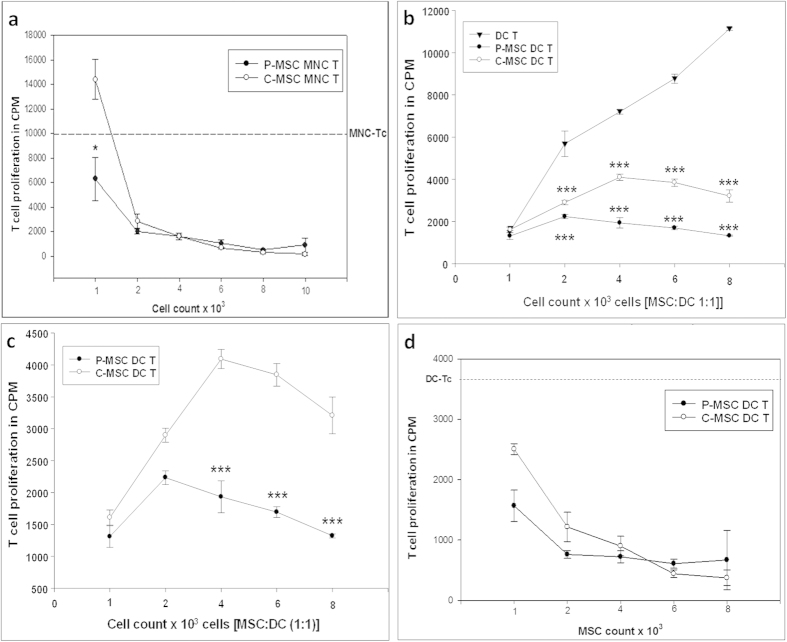
Effect of co-cultures of MNC/DC with P/C-MSCs on T cell proliferation: The T cell proliferation was assessed in terms of 3H thymidine incorporation. (**a**) The reduction in T cell proliferation in P-MSC/C-MSC co-cultures with PB-MNCs, (**b**) significant reduction in T cell proliferation in varying concentrations of DCs co-cultured in 1:1 ratio with P-/C-MSCs where (**c**) the reduction of T-cell proliferation was statistically significant in the presence of P-MSCs as compared to C-MSCs at higher cell concentrations and (**d**) similar reduction was observed with 10^4^ fixed number of DCs cultured with increasing number of P-/C-MSCs. In all cultures, there was a reduction in T cell proliferation in presence of MSCs and the reduction by P-MSCs was more pronounced than C-MSCs [n = 3, N = 3].

MSCs are known to have modulating effects on cells of the immune system. It has been reported that in co-cultures they hamper the antigen presenting ability of the cells thereby affecting the stimulation of T cells. Here, MSCs were seeded at different cell concentrations where the ratio of MSC:PB-MNCs (peripheral blood MNCs) was reduced. The co-culture was performed for 48 hrs and then 10^5^ T cells were seeded at constant cell number in all wells. Both P-MSCs as well as C-MSCs were effective in reducing the T-cell proliferation. However, a significant reduction was evident only in P-MSCs at the lowest cell dose [Fig. 2a]. The reduction shown by P-MSCs was about 1.4 fold as compared to MNC control and 2.1 fold as compared to C-MSCs.

### Placenta- and umbilical cord-derived MSCs differ in their ability to suppress the proliferation of T cells by dendritic cells

Dendritic cells (DCs) are excellent antigen presenting cells, which form an important mediator of innate and adaptive immune response and are capable of mounting an efficient T cell response for specific antigens. Dendritic cells were generated from human umbilical cord blood by a two-step culture method^24^. These DCs were characterized based on their morphology and marker expression profile [Supplementary Fig. S2] prior to co-culture with MSCs. They showed typical dendrites and were positive for markers like CD11c, MHC class I molecule HLA ABC, MHC class II molecule HLA DR, co-ctimulatory molecules CD80, CD86, CD83, CD40 and adhesion molecules like CD54 and CD58. DCs were co-cultured at different cell concentrations with either P-MSCs or C-MSCs in 1:1 ratio. Reduction in the T cell proliferation was seen in the MSC-DC-MLR as compared to only DC-MLR controls. Significant reduction in T cell proliferation was observed in case of both P-MSC and C-MSC co-cultures [Fig. 2b]. However, 50% reduction in the T cell proliferation was observed in P-MSC as compared to C-MSC co-cultures [Fig. 2c]. When a fixed number of dendritic cells was co-cultured with increasing number of MSCs, a reduction in the T cell proliferation was observed with increased MSC numbers [Fig. 2d]. This result indicates that immunosuppresive effect of P-MSCs on DCs is more pronounced as compared to C-MSCs.

### Conditioned medium of MSCs contains immunosuppressive activity

**Figure 3:**
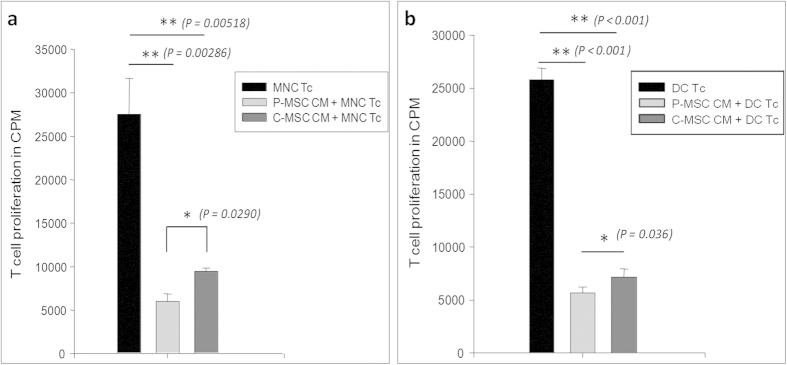
Reduction in T cell proliferation in MNC/DC cultures due to conditioned medium of P/C-MSCs: (**a**) MNCs and (**b**) DCs were cultured in the presence of 50% of P/C-MSC conditioned medium for 48 hrs. Cells were irradiated at 6400 rads. T cells were added to each well and allowed to proliferate. Proliferation was measured in terms of 3H thymidine incorporation. A significant reduction in T cell proliferation was observed in case of both MNC/DC cultures in presence of MSC CMs, which was more pronounced in P-MSC CM as opposed to C-MSC CM [n = 3, N = 2].

MSCs derived from both the sources affected the T cell proliferation ability of the antigen presenting cells in a co-culture system. So we wanted to see if similar kind of a difference is observed in case of cell free system i.e. by using conditioned medium (CM) of the MSCs. CM was prepared in plain medium over a confluent MSC culture and was collected after 48 hrs. 5 × 10^4^ PB-MNCs or 5 × 10^3^ or 10^4^ dendritic cells were cultured in 50% MSC-CM in IMDM with 10% FCS. MNCs/DCs in IMDM + 10% FCS without CM were kept as controls. T cells were then added in equal numbers to assess whether the CM is also efficient in affecting the ability of the APCs to stimulate T cell proliferation. We observed that in comparison to cells grown in control medium, P-MSC-CM and C-MSC-CM showed significant reduction in T cell proliferation where the difference in the effect exerted by P-MSCs was significant as compared to C-MSCs [Fig. 3a (MNCs) and b (DCs)]. Thus, cell-free extracts of both P-MSCs and C-MSCs also exert inhibitory effect on APCs thereby affecting the proliferation of T cells. We have observed the differences in the cytokine levels after keeping the cell numbers constant.

### P-MSCs produce better regulatory cytokine profile in MLR

**Figure 4:**
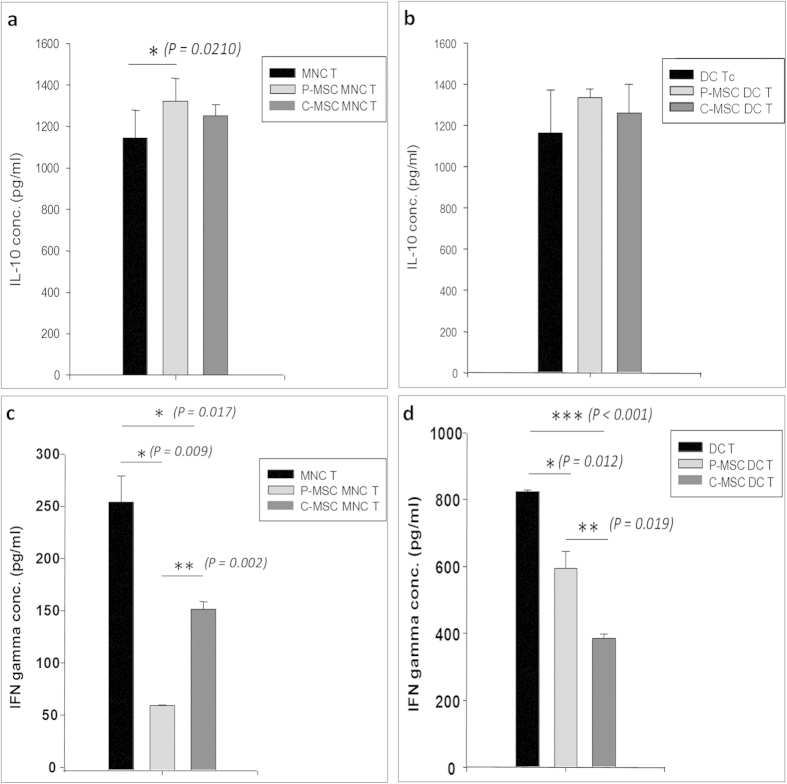
Secretion of immunoregulatory factors by MSCs in MLRs: P-MSCs and C-MSCs were co cultured with MNCs or DCs for 48 hrs after which T cells were added and kept for proliferation. Cell supernatants were collected at the end of culture period and analyzed for IL-10 and IFNγ. (**a**) Level of IL-10 was higher in MSC-MNC MLR as compared to MNC Tc alone controls with significant increase in case of P-MSC MNC MLR. (**b**) In MSC-DC MLRs, the IL-10 secretion in the co-culture was increased; however, it was not significantly higher. Similarly, IFNγ levels decreased significantly in presence of P-/C-MSCs in both (**c**) MSC-MNC and (**d**) MSC-DC MLRs. Significant decrease was seen in case of P-MSC vs C-MSCs in MSC MNC MLR [n = 3, N = 2].

A typical cytokine profile suggesting a regulatory phenotype involves the presence of IL-10 which leads to increase in pool of T regs. We measured the levels of IL-10, IFNγ and TGFβ1 in culture supernatants of MLRs after T cell proliferation, by ELISA. A regulatory profile was observed in case of our MSC co-cultures where the levels of IL-10 were higher and those of IFNγ were less as compared to controls, and an anti-inflammatory condition was attained. We observed increased levels of IL-10 in MLRs of P-MSCs where they were co-cultured with PB-MNCs [Fig. 4a] as well as with DCs [Fig. 4b]. The increase was significant in case of P-MSCs in MSC MNC MLRs compared to controls. Though no significance was observed in MSC-DC-MLRs, the increase in P-MSCs was evident as opposed to C-MSCs. A concomitant decrease in levels of IFNγ was also observed. IFNγ levels decreased significantly in presence of P-/C-MSCs in both MSC-MNC [Fig. 4c] and MSC-DC MLRs [Fig. 4d]. Significant decrease was seen in case of P-MSC vs C-MSCs in MSC MNC MLR.

### P-MSCs have a more regulatory milieu that supports increase in T-reg population in co-cultures as compared to C-MSCs

**Figure 5:**
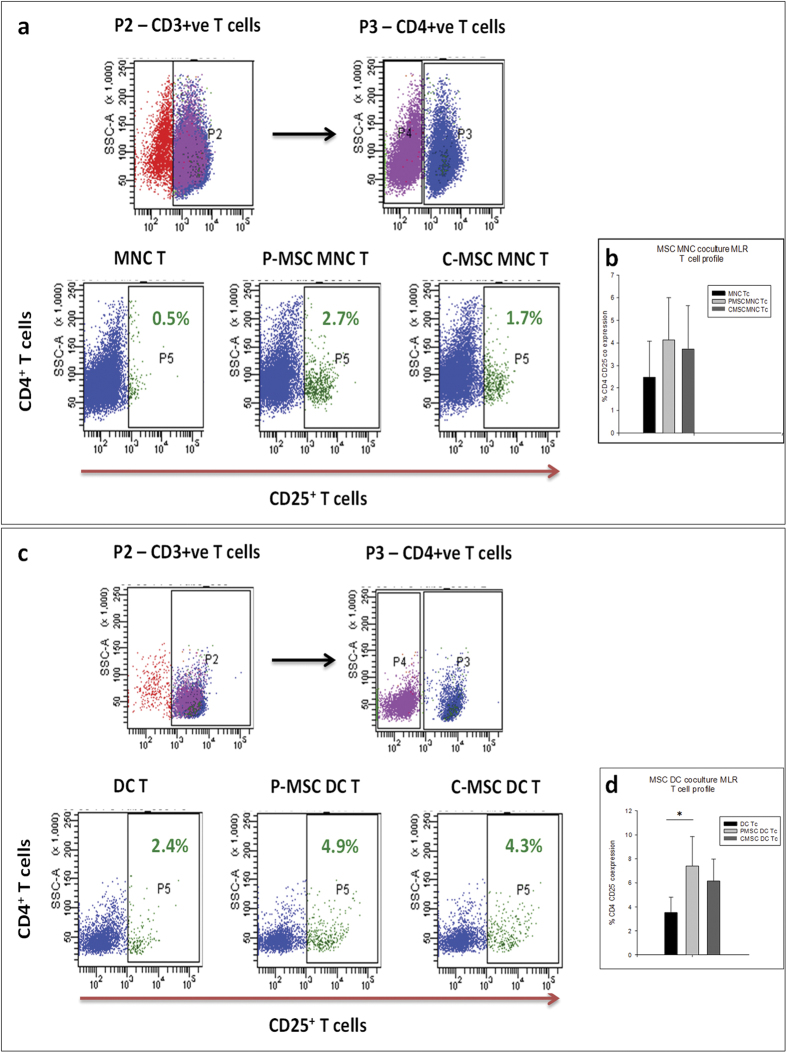
Increase in T reg population in MSC MLRs as detected by flow cytometry: T cells obtained after proliferation assay in MNC/DC co-cultures with P/C MSCs were harvested and stained with antibodies such as CD3 FITC, CD4 PE and CD25 APC. The CD3^+^ T cell population was gated and further analyzed for CD4 population on which the expression of CD25 was analyzed. (**a**) FACS profile of one representative sample is shown. There was increase in T reg population in cultures having P-/C-MSC-MNC. (**b**) However, the enhancement was not statistically significant as seen in graphical representation from data of 3 samples (**c**) Increase was also observed in P-/C-MSC-DC MLR cultures as compared to control. FACS profile of one representative sample. (**d**) Graphical representation of the same with data from three samples show that the increase was significant in P-MSC-DC-MLRs as compared to DC-T control (*P* *=* 0.026) [N = 3].

**Figure 6:**
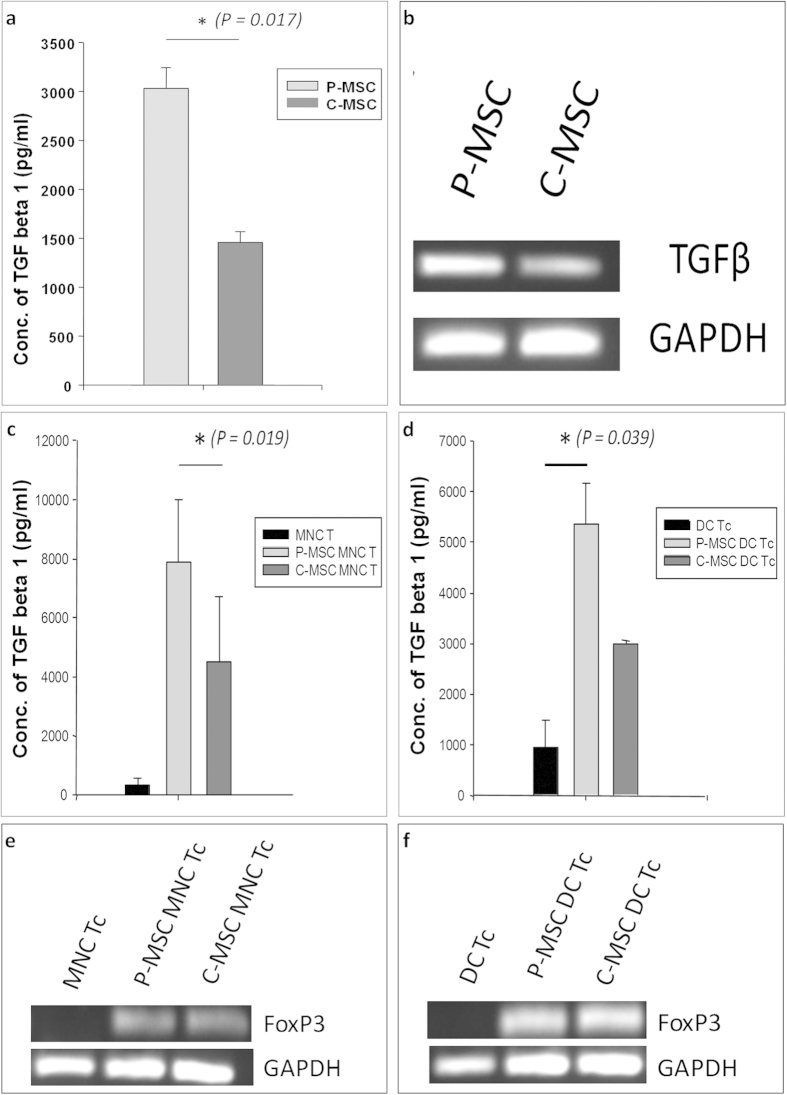
Increase in TGFβ levels and FoxP3 expression in MSC-MLRs: (**a**) Significant difference in TGFβ secretion in P-MSC CM as compared to C-MSC CM detected by ELISA (N = 3) and (**b**) difference in mRNA expression levels detected in P- and C-MSCs by semi-quantitative PCR (N = 3) (Gel image). P-MSCs and C-MSCs from the same donor were co-cultured with MNC/DCs. T cells were then allowed to proliferate on them. TGFβ levels in the CM, detected by ELISA [n = 3, N = 2], of (**c**) MSC MNC MLRs showing significant increase in P-MSC MLR CM as compared to C-MSC MLR CM and (**d**) in MSC DC MLRs where significant increase was observed in P-MSC MLR CM as compared to DC Tc control. Cells from the MLRs were harvested and the expression levels of FoxP3 transcript was detected in (**e**) MSC MNC MLR and (**f**) MSC DC MLR as compared to control (Gel image).

In order to check whether there was enrichment of T regs (T regulatory) in MSC APC MLRS, we checked the T cells (Tc) for the co expression of CD4 and CD25. We characterized the proliferated T cells in the co-cultures after the MLRs. Flow cytometry analysis was carried out where the cells were gated on CD3^+^ T cell subset. In the CD3+ fraction, the CD4 positive cells were gated. The CD25 positive cells were then assessed in the CD4+ cell fraction. We found that in both MNCs and DCs co-cultured with MSCs there was an increase in the CD4^+^ CD25^+^ cells [Fig. 5a,c]. The increase was apparent in case of P-MSCs as compared to C-MSCs in MSC MNC MLRs [Fig. 5b]. However, significant increase was found in P-MSC DC MLRs as compared to C-MSC DC MLRs [Fig. 5d]. These data suggest that the T cells attained a regulatory phenotype in case of MSC co-cultures. TGFβ – a cytokine implicated in the regulatory scenario was also assessed in P- and C-MSCs. Levels of TGFβ assessed by ELISA were significantly higher in P-MSC CM as compared to C-MSC CMs [Fig. 6a] and mRNA expression analysis reflected the same, showing higher expression in P-MSCs as compared to C-MSCs. [Figure 6b]. TGFβ1 was also found to be increased in MSC-MNC-MLR CMs [Fig. 6c] with a significant difference observed in P-MSCs as opposed to C-MSCs, as well as in MSC-DC-MLR CMs as compared to control [Fig. 6d]. Thus, P-MSCs imparted a better regulatory milieu as compared to C-MSCs in an allogenic MLR. We further went on to assess the mRNA expression levels of FoxP3 associated with T regs. We extracted total RNA from the harvested MLRs and checked for the expression of FoxP3, which was found to be increased in the P-MSC - MLRs as well as C-MSC - MLRs compared to control [Fig. 6e,f]. This supports the hypothesis that P-MSCs have an enhanced ability of supporting a modulatory phenotype.

## Discussion

MSCs are known to exhibit immunomodulatory properties which have been utilized in many cellular aspects. Due to their immuno-suppressive nature, they are known to support graft survival^10^. In view of these benefits, along with their rapid expansion ability, they have been employed in many clinical trials ( https://clinicaltrials.gov/). Though bone marrow is a reference standard for deriving MSCs, various other sources have been tapped for MSC isolation. Francisa Alcayaga-Martina *et al.* showed that menstrual fluid-derived MSCs were superior to BM-MSCs for HSC expansion^23^; while others showed a comparison of MSCs from different sources derived from healthy and diseased individuals to assess their efficacy^25^. They found that control MSCs were able to ameliorate the disease in lupus-prone mice while the lupus patient derived MSCs could not. Our study aims to assess the differences in MSCs that are derived from placenta and umbilical cord procured from same donor i.e. a similar *in vivo* environment. Though the umbilical cord is of purely fetal origin, the placental tissue is derived partly from the mother and partly from the developing fetus.

In this study our focus was on comparison of MSCs derived from paired placenta and umbilical cord tissue. Both P-MSCs and C-MSCs showed typical fibroblastic morphology, phenotypic marker expression and mesodermal lineage differentiation ability thereby fulfilling the criteria for MSCs as directed by the International Society for Cell Therapy^26^. Difference was seen in the clonogenic ability where the P-MSCs were able to give rise to higher number of CFU-Fs as compared to C-MSCs suggesting a higher expansion ability of P-MSCs. Thus, there is an advantage of working with P-MSCs as they will give sizable number of cells for transplantation purposes.

MSCs are implicated in modulating the immune system due to the absence of MHC class II molecule and co-stimulatory components such as CD80, CD86 and CD40. Dendritic cells (DCs) are important mediators of the innate and adaptive immune response capable of mounting an antigen-specific T cell response. Inhibition of the maturation and antigen-presenting functions of DCs derived from cord blood and monocytes by MSCs has been reported^27^,^28^. The inefficient priming of antigen presenting cells (APCs) leads to a reduced T cell proliferation^10^,^27^. When we assessed the immunomodulatory abilities of MSCs, we found that both P-MSCs as well as C-MSCs affected the proliferation of T-cells mediated by mononuclear cells as well as DCs. Though a difference in the decrease of T cell proliferation was not observed in MNC MLRs with higher cell numbers of P-MSCs and C-MSCs, we found a significant difference at the lowest concentration of P-MSCs suggesting that the P-MSCs in lower numbers would be still effective in their immunoregulatory property as compared to C-MSCs. The difference in case of P-MSCs and C-MSCs was significant in DC MLRs. The effect of MSCs is attributed to the cell to cell contact^29^ as well as to the secreted factors such as PGE2, IDO, IL-10, TGFβ^30^,^31^,^32^,^33^,^34^. Our data showed that both contact cultures (MSC-APC co-cultures) as well as non-contact cultures (50% MSC conditioned medium) of both the MSCs resulted in reduced T cell proliferation by MNCs and DCs. In both the cases, P-MSCs emerged out to be superior in their immunomodulation ability. This might be attributed to the fact that P-MSCs are a heterogeneous population comprising of cells of both the maternal and fetal combination leading to an additive effect in the action^35^. However, the fetal and maternal proportion of cells in the growing culture needs to be looked into.

On one hand where the soluble factors of MSCs grant the DCs a tolerogenic phenotype^27^, on the other hand they lead to T reg induction by reducing interleukin 4 (IL-4) and interferon γ (IFNγ) levels^28^,^36^,^37^. This is also associated with an increased IL-10 production imparting a regulatory phenotype to the T cells^36^,^38^. A similar effect was observed in case of our MSC co-cultures where the levels of IL-10 and TGFβ were higher and those of IFNγ were less as compared to controls where an anti-inflammatory condition was attained. MSCs are involved in the increase of T regs after transplantations^39^,^40^,^41^. In agreement to the previous findings, we found an increase in the CD4^+^ CD25^+^ T cell subset, where the expression of FoxP3 was also increased.

With all the benefits associated with MSCs, identification of sources for isolating the cells superior in all respects is still an aspect of study. In this report, we put forth P-MSCs as an alternative to C-MSCs for transplantation in case of immunological ailments and allogenic transplantation where the risk of graft rejection is high. Both BM-MSCs and C-MSCs are currently employed in many clinical trials. In a report where MSCs from healthy BM and UC were used, UC-MSCs were more effective in suppressing the IFNγ production compared to BM-MSCs^25^. IFNγ reduction and increased IL-10 shifts the balance from a pro-inflammatory to an anti-inflammatory state which is necessary for a regulatory phenotype in cases of transplantation. Placental tissue can be obtained with an equivalent frequency as the umbilical cord, and hence can be cultured and expanded with ease. In the current study, we report a novel finding that P-MSCs are superior in terms of their expansion ability and immune regulatory properties to that of C-MSCs. Thus, our findings present P-MSCs as a better substitute to C-MSCs in cellular transplantation.

## Materials and Methods

### Ethical approval

All protocols and methods for collection and processing of human samples like placenta, umbilical cord, cord blood and peripheral blood were approved by National Centre for Cell Science Ethics Committee (NCCS-IEC) and NCCS Committee for Stem Cell Research (NCCS-IC-SCR), which is in accordance with the Declaration of Helsinki. Prior informed consent was taken from the volunteers. The format of consent forms was also approved by the NCCS-IEC and NCCS-IC-SCR. The experiments were performed in triplicates with cells from one donor (n = 3). 3 different paired samples were investigated to confirm the findings (N = 3).

### MSCs from placenta and cord tissues

Placenta and umbilical cord were collected from full term deliveries. The protocol for MSC isolation is as described earlier^42^. Briefly, placenta and a piece of cord were washed in plain IMDM (Sigma Aldrich, St Louis, USA) and chopped mechanically. Enzymatic digestion was done and single cell suspension was further cultured in medium containing 20% mesenFBS (Gibco, Life Technologies, Grand Island, USA). MSCs were identified on the basis of their fibroblastic morphology and phenotypic characterization which was performed after passage 3 and was assessed by flow cytometry (BD Canto II, BD Biosciences). Single cell suspension of cells was made after trypsinizing the cells and staining for surface markers was done by performing dual staining using a panel of antibodies: CD105-PE + CD73-APC, CD166-PE + CD45-APC, HLA ABC-APC + HLA DR-PE and CD44-APC + CD34-PE. All antibodies were purchased from BD Pharmingen, CA, USA and CD90-APC and CD45-APC from eBioscience, San Diego, CA, USA. Mesodermal lineage differentiation towards the adipogenic, osteogenic and chondrogenic lineages was carried out using respective differentiation media (All from Gibco, Life Technologies, Grand Island, USA). The stains like oil red O, Alizarin red S and Alcian blue were used for identification of adipocytes, osteocytes and chondrocytes respectively after formaldehyde fixation (All stains were procured from Sigma Aldrich, St Louis, USA).

### Collection of Conditioned media

For preparation of MSC CMs, confluent MSC cultures were grown in plain medium for 48 hrs and the supernatants were collected and pelleted down to remove cells and debris.

### PB-MNCs

Peripheral blood was diluted in plain IMDM. Mononuclear cells (PB-MNCs) were isolated by density gradient centrifugation using Ficoll-hypaque (HiMedia, India).

### DC generation from cord blood

DCs were expanded from cord blood derived mononuclear cells by a two step culture system established in our lab^24^. Briefly, MNCs were subjected to 1 hr plastic adherence to remove the monocytes. The non adherent population was cultured for expanding the CD34+ cells towards enrichment of DC progenitors using growth factors like Flt3L, SCF and TPO for 3 weeks. The cells were then subjected to differentiation using GM-CSF and IL3 for 3 days, GM-CSF and TNFα for 4 days. These cells were then pulsed with lipopolysaccharide for maturation. DCs were characterized on the basis of their morphology and by a panel of antibodies for positive marker expression like CD11c, CD83, CD80, CD86, CD1a, CD40, MHC class I and class II molecules by flow cytometry.

### CFU-F assay

5 × 10^3^ or 10^4^ MSCs were seeded per plate in 60 mm dishes in triplicates and incubated for 7–10 days. The monolayer was washed, fixed in methanol and stained with 0.1% crystal violet solution for 10 min. Clones of more than 50 cells were scored as a colony forming unit – fibroblast.

### MLR in co-cultures of MNCs/DCs with MSCs

MSCs were plated in 96-well plates (BD Bioscience) at different cell concentrations in triplicates and cultured for 24 h. PB-MNCs (5 × 10^4^/well) were added and co-cultured with MSCs for 48 hrs. Similarly for MSC-DC co-cultures, DCs were co-cultured with MSCs in 1:1 ratio for 48 hrs. MNCs or DCs without MSCs were kept as positive control.

In CM experiments, MNCs and DCs were cultured in 50% MSC-CMs for 48 hrs.

All cultures were irradiated at 6400 rads prior to the addition of T cells.

For an MLR, T cells were obtained from peripheral blood by using RosetteSep T cell enrichment cocktail (Stem Cell Technologies Inc., Vancouver, British Columbia, Canada) as per manufacturer’s instructions. 10^5^ T-cells were added to each well. Co-cultures without T cells were kept as negative controls. T cell proliferation was quantitated by 3H thymidine (BRIT, Mumbai) uptake assay.

### Cytokine content analysis

Cytokine levels in supernatants of the MLRs were analysed by commercially available OptEIA ELISA kits for hIL-10, hIFNγ and hTGFβ1 (purchased from BD Pharmingen, CA, USA) according to the manufacturer’s instructions. The experiment was performed in triplicates with cells from one donor (n = 3) and 2 different paired samples [N = 2]. The P- and C-MSCs were seeded in equal numbers separately, co-cultured with equal numbers of MNCs or DCs for 48 hrs. The T-cells were also added at the same time point in equal numbers to all the wells.

### Assessment of T-cell regulatory profile

T cells were collected from the cell suspensions of MLR, washed with PBS and incubated with antibodies against CD3-FITC, CD4-PE and CD25-APC for 45 min on ice. Cells were fixed and flow cytometric analysis was performed using BD FACS Canto II (BD Biosciences).

### RNA isolation and Semi-quantitative PCR

Total RNA was prepared from T-cells using Trizol reagent (Invitrogen, USA). 1 μg RNA was transcribed into cDNA using MMLV reverse transcriptase (Invitrogen, USA) and 150 ng of random primers (Invitrogen, USA). Primer sequences for GAPDH, FoxP3 and TGFβ are shown in Table 1.

### Statistical analysis

This was carried out using SigmaStat software by performing ANOVA. As multiple comparisons were done – ANOVA was used. All data are presented graphically with error bars as mean ± SEM, and P ≤ 0.05 was considered statistically significant.

## Additional Information

**How to cite this article**: Talwadekar, M. D. *et al.* Placenta-derived mesenchymal stem cells possess better immunoregulatory properties compared to their cord-derived counterparts - a paired sample study. *Sci. Rep.* **5**, 15784; doi: 10.1038/srep15784 (2015).

## Supplementary Material

Supplementary Information

## Figures and Tables

**Table 1:** Sequences of primers for semi-quantitative PCR.

No.	Gene name	Sequence (5′ → 3′)
1	Human GAPDH forward	CGG ATT TGG TCG TAT TG
2	Human GAPDH reverse	GGA AGA TGG TGA TGG GA
3	Human TGFβ1 forward	CAC AAC GAA ATC TAT GAC AA
4	Human TGFβ1 reverse	GGT TGC TGA GGT ATC GC
5	Human FoxP3 forward	TTC GAA GAG CCA GAG GAC TT
6	Human FoxP3 reverse	ATG GCA CTC AGC TTC TCC TT
