# Supplementary material for: Placenta-derived mesenchymal stem cells possess better immunoregulatory properties compared to their cord-derived counterparts–a paired sample study
Source: Sci Rep. 2015 Oct 28;5:15784. doi: 10.1038/srep15784 (PMC4623529; doi:10.1038/srep15784)

**Placenta-derived mesenchymal stem cells possess better immunoregulatory properties compared to their cord-derived counterparts – a paired sample study.**

Manasi D. Talwadekar^1^, Vaijayanti P. Kale^1^ and Lalita S. Limaye^1^,*

^1^Stem Cell Laboratory, National Centre for Cell Science, NCCS Complex, University of Pune Campus, Ganeshkhind, Pune, India

*****Dr. Lalita Limaye; email address: lslimaye@nccs.res.in

**Supplementary Fig S1.** Mesodermal lineage differentiation shown by placenta- and cord-derived MSCs stained with (a & b) Oil Red O for adipocytes , with (c & d) Alizarin Red S for osteocytes and with (e & f), Alcian blue for chondrocytes respectively.

**Supplementary Fig S2.** Characterization of dendritic cells cultured from umbilical cord blood. (a) DC profile on flow cytometer (b) Typical DC morphology showing dendrites in phase contrast (c) Phenotypic marker analysis of DCs assessed by expression of markers like CD11c, MHC class I and II molecules HLA ABC and HLA DR, co-stimulatory molecules CD86, CD80, CD40, CD83 and adhesion molecules like CD58 and CD54.


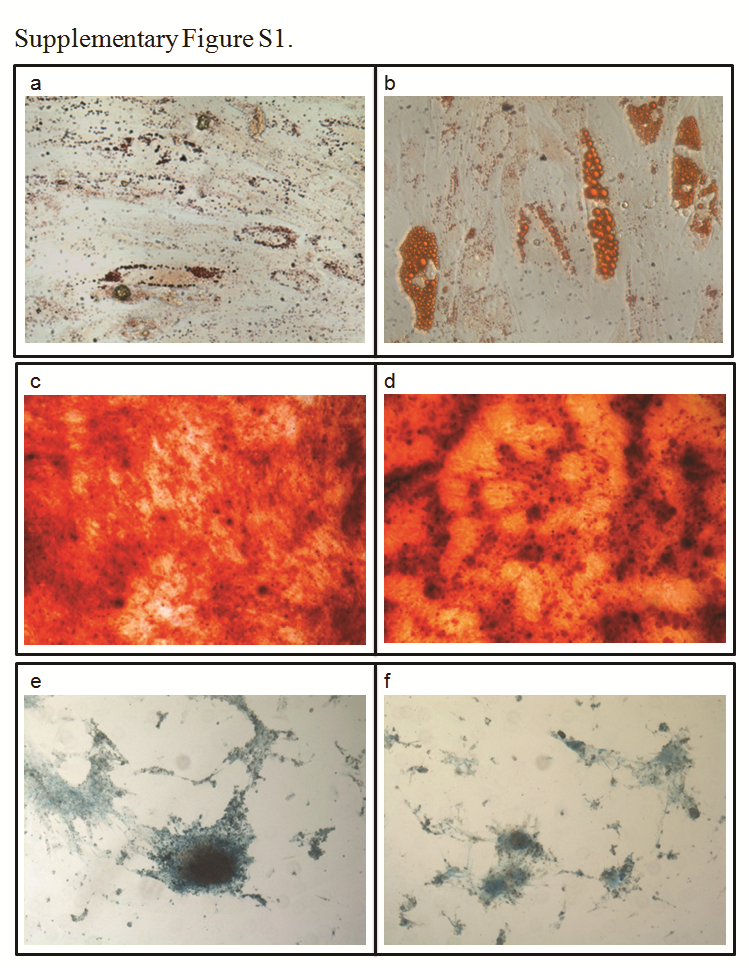


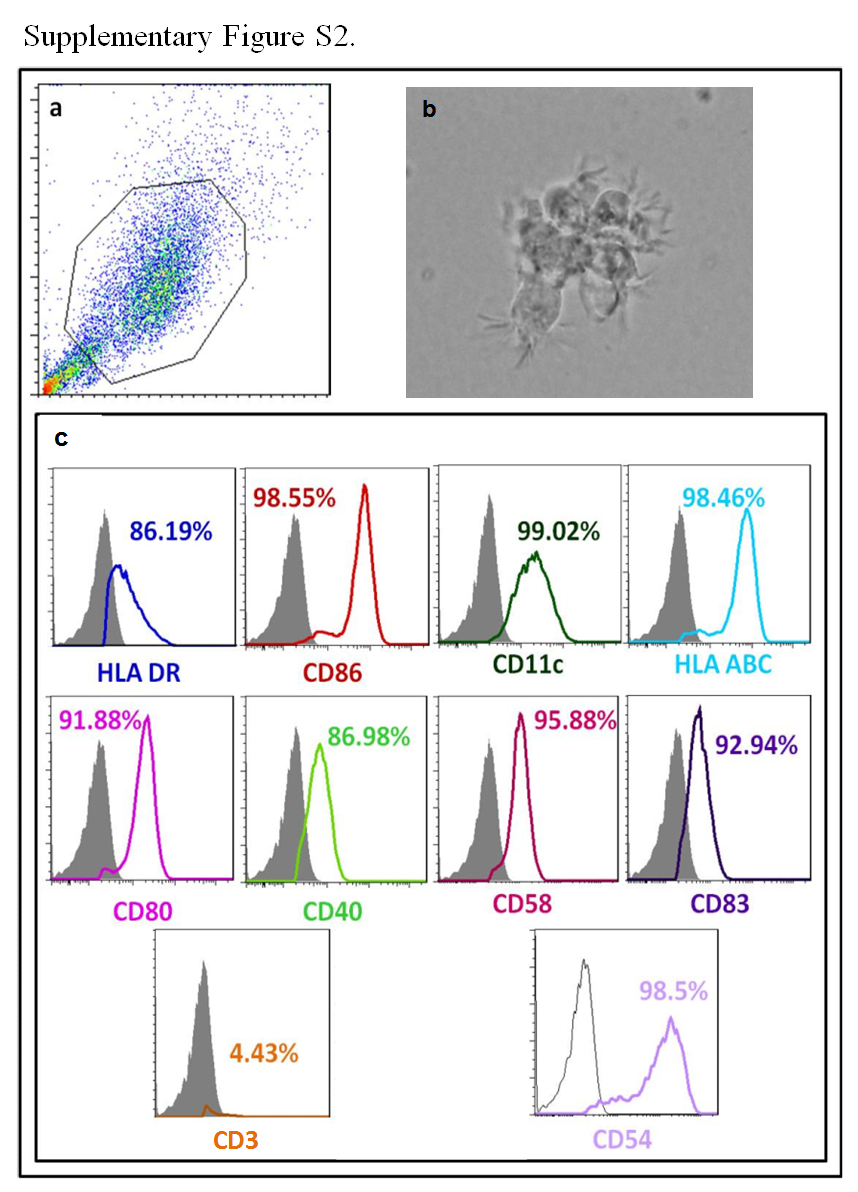

Supplement: Supplementary Information [file srep15784-s1.doc]
